# A review of European and international phthalates regulation: focus on daily use products: Marco Monti

**DOI:** 10.1093/eurpub/ckac131.226

**Published:** 2022-10-25

**Authors:** M Monti, M Fasano, L Palandri, E Righi

**Affiliations:** 1 Postgraduate School of Public Health, University of Modena and Reggio Emilia, Modena, Italy; 2 Department of Biomedical Sciences, University of Modena and Reggio Emilia, Modena, Italy

## Abstract

**Background:**

Phthalates are known endocrine disruptors used in a wide range of industrial and household products. With globalization and interdependency of the supply chain, the control of toxic substances in daily use products has become more challenging. Many countries have implemented laws and policies to limit their use, although these regulations are neither unified nor seem adequate, as studies suggest that more vulnerable populations (children, pregnant women) are exposed to phthalates that should be restricted.

**Methods:**

For seven of the most used phthalates - bis(2-Ethylhexyl) phthalate (DEHP), Butylbenzyl phthalate (BBP), Dibutyl phthalate (DBP), Diisobutyl phthalate (DIBP), diisononyl phthalate (DINP), diisodecyl phthalate (DIDP), Di-n-octyl phthalate (DNOP) - we performed an online research on institutional sites and dedicated Agencies of the three largest world economies (European Union (EU), United States of America (USA), China) to analyze their uses and bans, focusing on Food Contacts Materials (FCM), cosmetics, toys and childcare articles.

**Results:**

In the EU area DEHP, BBP, DBP and DIBP are not allowed in toys and childcare articles above 0,1% by weight. All the seven phthalates are also severely restricted in FCM, and mostly banned as cosmetic components. In the USA, there is no formal prohibition to their use in cosmetics but phthalates are mostly limited in FCM. In China, the limit for DBP, BBP, DEHP, DNOP, DINP, DIDP in plastic toys is 0,1% of the material composition; regarding cosmetics DEHP, BBP and DBP are prohibited.

**Conclusions:**

We found substantial differences in the international legislation. Though there is essential agreement on toys and childcare articles legislation, there are many discrepancies about FCM and cosmetics. Further research is needed to compare the regulation with data about concentrations of these ubiquitous elements, to underline the real exposure and risk in different populations and to improve knowledge and safety on this matter.

**Key messages:**

• Phthalates, known endocrine disruptors, in daily use products are a matter of concern.

• Coordinated international laws to prevent exposure, especially in vulnerable populations, are needed.

